# Microbiome–Immune Interactions as Determinants of Checkpoint Inhibitor Efficacy in Hepatocellular Carcinoma

**DOI:** 10.3390/ijms27177543

**Published:** 2026-08-23

**Authors:** Madalina Raluca Ostafe, Simona Ruxandra Volovat, Ana Clement, Cezara Ioana Litcanu, Smaranda Iuliana Tabarcea, Cristian Constantin Volovat, Diana-Ioana Panaite, Iolanda Georgiana Augustin, Constantin Volovat

**Affiliations:** 1 Department of Medical Oncology-Radiotherapy, “Grigore T. Popa” University of Medicine and Pharmacy, 700115 Iasi, Romania; cezaraioanali@gmail.com (C.I.L.); dianaiboboc@gmail.com (D.-I.P.); volovat.constantin@umfiasi.ro (C.V.); 2 Regional Institute of Oncology, 700483 Iasi, Romania; clementana19@gmail.com (A.C.); smaranda-iuliana.tabarcea@email.umfiasi.ro (S.I.T.); 3 Department of Radiology, “Grigore T. Popa” University of Medicine and Pharmacy, 700115 Iasi, Romania; cristian-constantin.volovat@umfiasi.ro; 4 Department of Oncology, “Carol Davila” University of Medicine and Pharmacy, 050474 Bucharest, Romania; iolanda.augustin@gmail.com

**Keywords:** hepatocellular carcinoma, gut microbiota, gut–liver axis, immunotherapy, immune checkpoint inhibitors, dysbiosis, microbiome, bile acids, fecal microbiota transplantation, precision oncology

## Abstract

Hepatocellular carcinoma (HCC) remains a major global health challenge and one of the leading causes of cancer-related mortality, with advanced disease continuing to be associated with limited therapeutic options and substantial heterogeneity in response to systemic treatment. Recent evidence has established the gut microbiota, through the gut–liver axis, as a critical determinant of immunotherapy efficacy, while also influencing antitumor immunity and liver carcinogenesis. Microbial dysbiosis may promote chronic inflammation, intestinal barrier disruption, bacterial translocation, and immune dysfunction, thereby contributing to hepatocarcinogenesis. Moreover, gut microbial composition and microbial-derived metabolites, including bile acids, short-chain fatty acids (SCFAs), and inosine, have been associated with modulation of antitumor immune responses and differential outcomes to immune checkpoint inhibitors (ICIs). Emerging clinical evidence in HCC has identified distinct gut microbial signatures associated with response to nivolumab, pembrolizumab, and atezolizumab-based regimens, including enrichment of *Akkermansia muciniphila* and SCFA-producing taxa such as *Ruminococcaceae*, *Roseburia*, and *Prevotella* in responders. However, these findings remain inconsistent across studies, with no reproducible microbial signature identified because of small cohort sizes, heterogeneous patient populations, geographic variation, cirrhosis-related confounding factors, and methodological differences in microbiome analysis. This review summarizes the current understanding of microbiome–immune interactions in HCC, examines mechanistic pathways linking the microbiota to immunotherapy response, critically evaluates available clinical evidence, and discusses current limitations and future therapeutic strategies, including fecal microbiota transplantation, probiotics, dietary modulation, and engineered bacterial platforms. Collectively, microbiome-based approaches may contribute to the development of personalized immunotherapeutic strategies in HCC, although larger standardized prospective studies are required before microbiome-derived biomarkers can be implemented in routine clinical practice.

## 1. Introduction

Hepatocellular carcinoma (HCC) accounts for approximately 85–90% of primary liver cancers and remains the third leading cause of cancer-related mortality worldwide. Despite advances in surveillance and treatment, outcomes remain poor, with a 5-year overall survival rate below 30% and curative options applicable to only a minority of patients. HCC most commonly arises in the setting of cirrhosis, traditionally driven by viral hepatitis, although the global disease burden is increasingly shifting toward alcohol-related liver disease and metabolic dysfunction [1]. While early-stage disease may be amenable to curative strategies such as resection, transplantation, or locoregional therapies, advanced HCC relies primarily on systemic treatments, including targeted therapies and immune checkpoint inhibitors (ICIs). Although immunotherapy has improved clinical outcomes in recent years, response rates remain suboptimal, and substantial interpatient variability persists, highlighting the urgent need for more effective therapeutic strategies and robust predictive biomarkers [2].

This variability is partly rooted in the unique immunological landscape of HCC. Unlike most solid tumors that develop within relatively stable immune environments, HCC arises in the context of chronic liver injury and cirrhosis, conditions characterized by persistent inflammation, immune dysfunction, and impaired immune surveillance [3]. Within this setting, the liver operates as a highly specialized immunological organ, constantly exposed to antigens derived from the gut through the portal circulation [4]. This anatomical and functional connection forms the basis of the gut–liver–immune axis, a complex network involving bidirectional interactions between the intestinal microbiota, hepatic cells, and both innate and adaptive immune compartments [5]. Continuous exposure to microbial-associated molecular patterns (MAMPs), metabolites, and dietary antigens requires a tightly regulated balance between immune tolerance and activation, a balance that becomes disrupted in chronic liver disease [6].

In this context, the gut microbiome has emerged as a key modulator of both tumor biology and host immune responses. Increasing evidence indicates that microbial composition and function can influence not only carcinogenesis but also tumor progression [7] and response to therapy [8]. Given its intimate interaction with the immune system—particularly within the liver—the microbiome represents a dynamic and potentially modifiable factor that may contribute to the heterogeneity of immunotherapy outcomes. This raises the possibility that targeting the microbiome could enhance therapeutic efficacy and open new avenues for precision oncology in HCC.

This review aims to evaluate the role of the gut microbiome as a determinant of response to ICIs in HCC. We integrate current evidence from preclinical models, studies in other solid tumors, and emerging clinical data in HCC to explore the underlying biological mechanisms and their potential clinical implications. Understanding these interactions requires first an appreciation of the distinctive immunologic environment in which HCC develops, which forms the foundation for the complex interplay between tumor, host immunity, and microbiome.

## 2. The Unique Immunologic Context of HCC

The liver represents a unique immunological organ that continuously integrates signals from both the systemic circulation and the gut, resulting in a finely tuned balance between immune tolerance and inflammation. It harbors an intricate network of resident immune cells—including Kupffer cells, dendritic cells, natural killer (NK) cells, and various lymphocyte subsets—that operate in close coordination with non-immune components such as hepatocytes and liver sinusoidal endothelial cells. This highly organized cellular architecture enables constant immune surveillance while preserving tolerance to non-threatening antigens originating from the gut [9]. As a frontline interface between the gastrointestinal tract and the systemic circulation, the liver is continuously exposed to a diverse array of nutrients, metabolites, and microbe-derived signals via the portal vein. To cope with this persistent antigenic load, hepatic immune responses are tightly regulated, favoring controlled activation rather than sustained inflammation [10].

**Figure 1:**
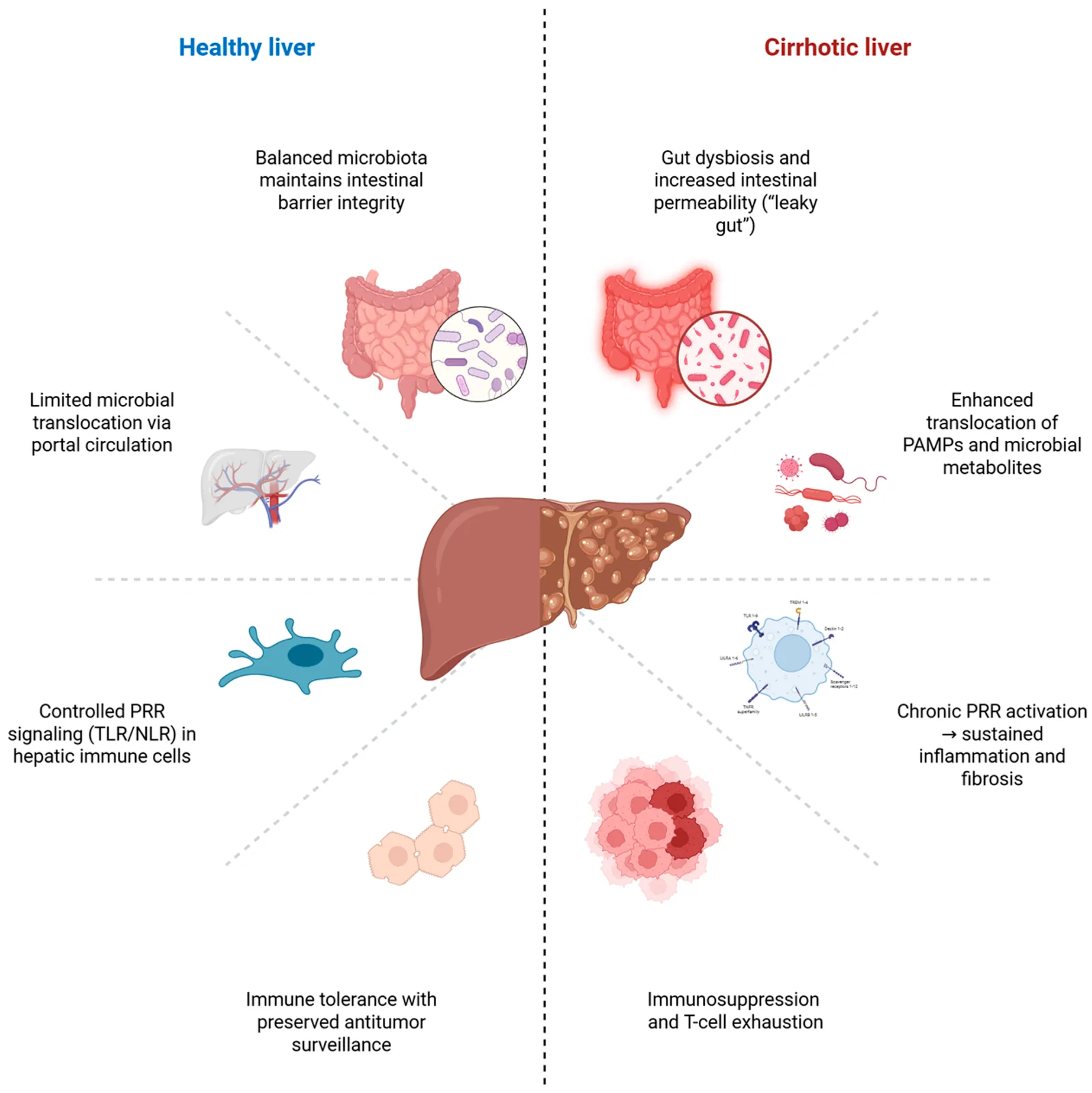
Gut–liver axis alterations in healthy and cirrhotic liver states. In healthy conditions, a balanced gut microbiota preserves intestinal barrier integrity, limits microbial translocation through the portal circulation, and maintains controlled pattern recognition receptor (PRR) signaling in hepatic immune cells, supporting immune tolerance while preserving antitumor surveillance. In cirrhosis, gut dysbiosis and increased permeability promote enhanced transfer of pathogen-associated molecular patterns (PAMPs) and microbial metabolites to the liver, leading to chronic PRR activation, sustained inflammation, fibrosis, immunosuppression, and T cell exhaustion. Created in BioRender. Ostafe, M. (2026) https://www.biorender.com/7kmk8sj.

Consequently, instead of mounting strong inflammatory reactions to all incoming stimuli, the liver promotes a predominantly tolerogenic environment in which harmless antigens are effectively ignored, while retaining the capacity to respond to genuine threats. This balance is essential for maintaining immune homeostasis, preventing unnecessary tissue damage, and supporting overall metabolic and immunological stability [11]. However, this intrinsic bias toward immune regulation also creates a permissive background in which pathological processes may evolve under conditions of chronic stress [7]. In contrast to acute inflammation, which is self-limiting and resolves following tissue repair, chronic liver inflammation persists without adequate resolution, thereby promoting a tumor-permissive environment. This sustained inflammatory state drives continuous cellular stress and regenerative pressure, facilitating the accumulation of genetic alterations [12]. At the same time, hepatic metabolism actively shapes immune cell function through pathways such as Toll-like receptor signaling and inflammasome activation, linking metabolic dysregulation to inflammation and tumor progression (Figure 1) [13].

A hallmark of HCC development is the coexistence of persistent inflammatory signaling with a progressively impaired immune response. In this setting, long-standing inflammation—commonly driven by viral hepatitis, alcohol-related liver disease, or metabolic dysfunction—generates a microenvironment characterized by ongoing cellular injury, regeneration, and genetic instability [13]. Continuous exposure to pathogen-associated and damage-associated molecular patterns sustains activation of innate immune pathways, leading to hepatocyte damage, compensatory proliferation, and fibrogenesis, all of which contribute to hepatocarcinogenesis [14]. This close interplay between metabolic and immune processes represents a defining feature of liver pathology and distinguishes HCC from many other solid tumors [15]. Given the liver’s constant exposure to gut-derived metabolites and microbial signals, this immune–metabolic landscape is inextricably linked to the gut–liver axis, which emerges as a central framework for understanding how external factors, including the microbiome, may influence immune regulation and tumor progression in HCC.

## 3. Mechanistic Links Between the Gut Microbiome and Antitumor Immunity in HCC

The gut microbiome is increasingly recognized as a critical regulator of host physiology, with essential roles in metabolism, immune homeostasis and inflammatory signaling. Its influence on immune function has driven growing interest in the interplay between microbiota and anticancer immunity, particularly in the context of ICIs [16]. Clinical and preclinical data indicate that microbiome composition is associated with differential responses to ICIs, enabling stratification of patients into responders and non-responders [17]. However, the underlying mechanisms remain incompletely defined, reflecting heterogeneity in study design and patient populations. Current evidence suggests that microbiome-mediated modulation of antitumor immunity is not governed by a single pathway, but rather by a multilayered network involving innate immune sensing, metabolic signaling, and tumor microenvironment (TME) interactions.

### 3.1. Dysbiosis, Intestinal Barrier Dysfunction, and Microbial Translocation

The intestinal barrier is a complex, multilayered defense network composed of a tightly regulated epithelial lining, overlying mucus, antimicrobial peptides, and specialized immune structures within the gut-associated lymphoid tissue [18]. Its integrity is dynamically regulated by microbial metabolites, such as short-chain fatty acids, which support epithelial function through G-protein-coupled receptors [19]. Secretory immunoglobulin A provides an additional layer of protection by limiting bacterial invasion and restricting the passage of luminal antigens into the systemic circulation [20]. The resident microbiota also contributes to barrier homeostasis by promoting epithelial renewal and mucin production, competing with pathobionts for nutrients and ecological niches, as well as suppressing potentially pathogenic species through bacteriocin production [21].

**Figure 2:**
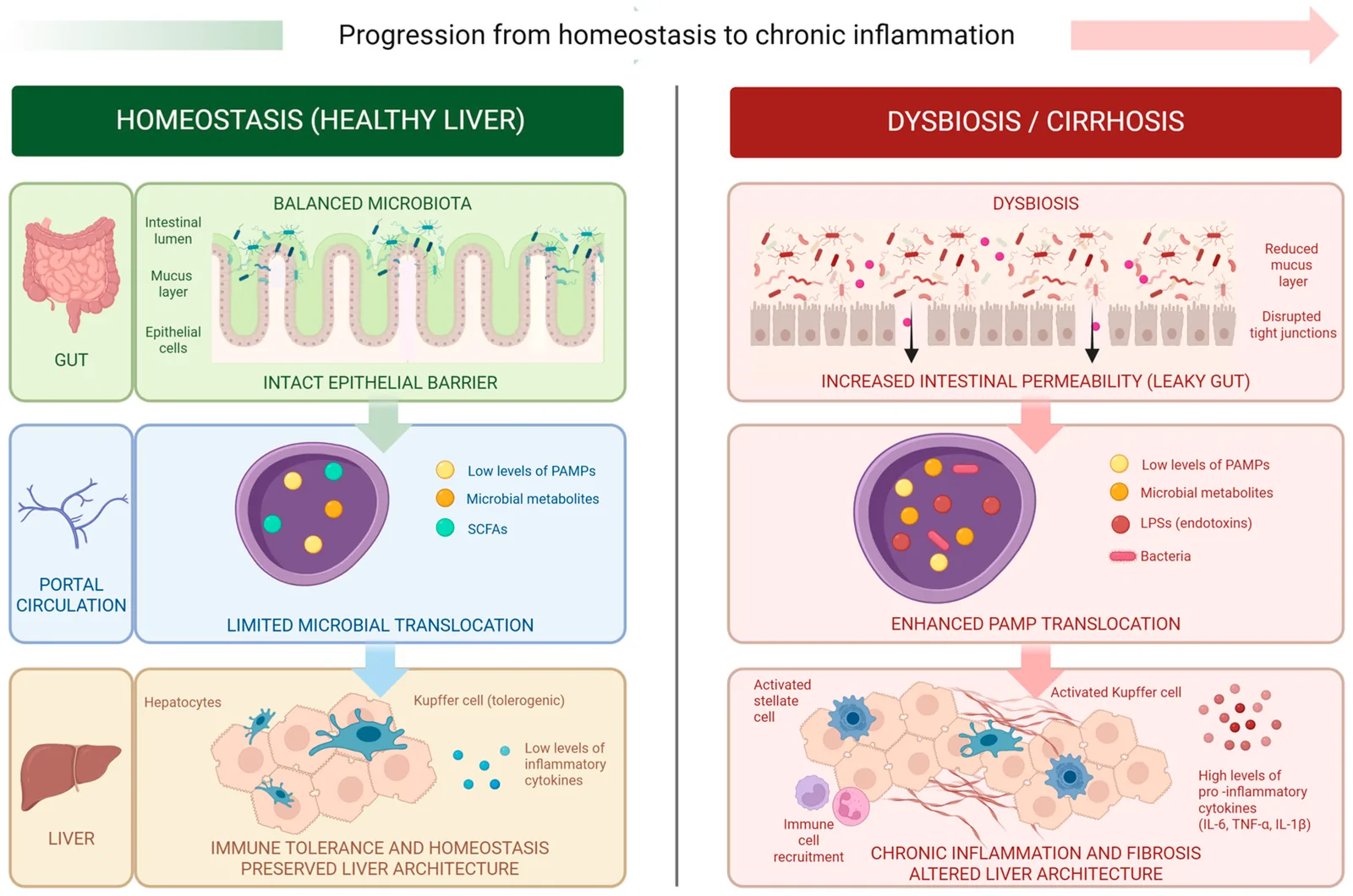
The progression from gut–liver homeostasis to chronic inflammation and cirrhosis. In physiologic conditions, a balanced gut microbiota and a healthy intestinal barrier limit microbial translocation via portal circulation, maintaining the immune tolerance of the liver and preserving liver architecture. In contrast, dysbiosis disrupts epithelial integrity and increases intestinal permeability, promoting translocation of bacteria, LPSs, and other pathogen-associated molecular patterns (PAMPs) to the liver. Persistent activation of Kupffer cells and hepatic stellate cells induces pro-inflammatory cytokine production, immune cell recruitment, chronic inflammation, fibrosis, and progressive alteration of liver architecture. Created in BioRender. Ostafe, M. (2026) https://BioRender.com/xngc938.

In the context of dysbiosis—defined as an imbalance in microbial composition and function—this barrier becomes compromised, leading to increased intestinal permeability (“leaky gut”). As a result, bacterial products such as lipopolysaccharide, peptidoglycans, and bacterial DNA can translocate into the portal circulation. This process, often referred to as metabolic endotoxemia, represents a key driver of hepatic immune activation and systemic inflammation (Figure 2) [22].

In chronic liver disease, particularly cirrhosis, disruption of these protective mechanisms results in increased intestinal permeability and enhanced translocation of microbial products into the portal circulation [23]. Consequently, the liver becomes persistently exposed to pathogen-associated molecular patterns (PAMPs), which engage pattern recognition receptors (PRRs) such as Toll-like receptors (TLRs). While controlled exposure to microbial signals contributes to immune homeostasis, persistent translocation drives sustained immune activation, promoting hepatic inflammation and immune dysregulation. This altered microbial exposure reshapes the hepatic immune landscape, favoring tumor-promoting inflammation while impairing effective antitumor immunity. In this context, commensal microorganisms may support immune competence, whereas pathobionts can suppress cytotoxic immune responses, underscoring the dual and context-dependent role of the microbiome in cancer progression [24].

Recent evidence provides direct support for intestinal barrier disruption as a functional driver of hepatocarcinogenesis. Fecal microbiota transplantation from patients with HCC into germ-free or specific-pathogen-free mice impaired intestinal barrier integrity and reduced colonic mucus thickness and tight-junction protein expression, while facilitating the translocation of live bacteria to the liver. These alterations were accompanied by hepatic inflammation, fibrosis, dysplasia, and accelerated HCC development. Among the translocating microorganisms, Klebsiella pneumoniae emerged as a principal driver: monocolonization produced barrier injury and hepatocarcinogenesis, while its surface protein PBP1B (penicillin-binding protein 1B) activated TLR4-dependent inflammatory and oncogenic signaling in HCC cells [25]. Complementary experimental evidence indicates that dysbiosis and bacterial translocation promote TLR4-dependent expansion of hepatic monocytic myeloid-derived suppressor cells and depletion of CD8^+^ (cluster of differentiation) T cells, whereas restoration of *Akkermansia muciniphila* improves barrier function and attenuates hepatic inflammation and fibrosis [26].

Collectively, these findings highlight intestinal barrier disruption as a central event linking dysbiosis to chronic hepatic inflammation and impaired antitumor immunity. However, the biological consequences of microbial translocation ultimately depend on how these signals are sensed and interpreted by hepatic immune cells. This next level of regulation is mediated through innate immune recognition pathways, which shape downstream immune activation, tolerance, and immune priming within the liver microenvironment.

### 3.2. Innate Immune Sensing and Microbiota-Driven Immune Reprogramming

**Figure 3:**
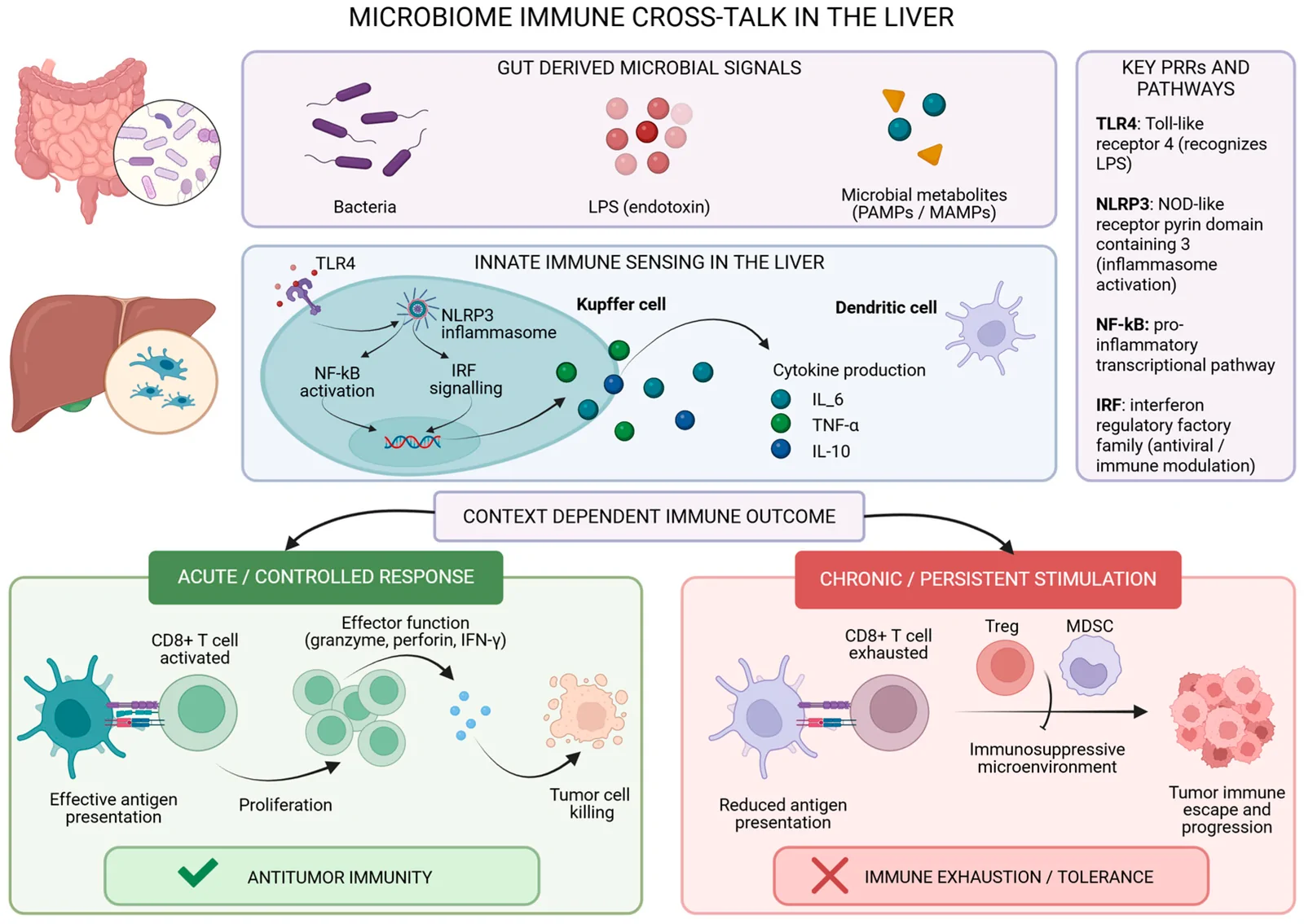
Microbiome–immune crosstalk in the liver. Gut-derived microbial signals, including bacteria, LPS, and MAMPs, activate hepatic innate immune pathways through pattern recognition receptors such as TLR4 and the NLRP3 inflammasome. Subsequent activation of NF-κB and IRF signaling in dendritic and Kupffer cells modulates cytokine production and shapes the hepatic immune microenvironment. The immune outcome is context dependent. Acute or controlled stimulation promotes effective antigen presentation, CD8^+^ T cell activation, and antitumor immunity, whereas chronic stimulation induces immune exhaustion, accumulation of immunosuppressive cells, and tumor immune escape, ultimately favoring hepatocellular carcinoma progression. Created in BioRender. Ostafe, M. (2026). https://BioRender.com/vn4v6bz.

Within the liver, Kupffer cells, liver sinusoidal endothelial cells, hepatic stellate cells, dendritic cells, and innate lymphoid populations coordinate responses to gut-derived microbial signals [27]. Kupffer cells serve as principal hepatic sentinels through PRRs, particularly TLRs and nucleotide-binding oligomerization domain-like receptors [12]. Recognition of lipopolysaccharide by TLR4 activates MyD88 (myeloid differentiation primary response 88)- and TRIF (Toll/interleukin-1-domain-containing adapter-inducing interferon-β)-dependent pathways, with downstream engagement of NF-κB (nuclear factor kappa-light-chain-enhancer of activated B cells), interferon regulatory factors, and STAT3 (signal transducer and activator of transcription 3). These signaling cascades induce TNF-α (tumor necrosis factor alpha), IL (interleukin)-1β, and IL-6 production, recruit inflammatory cells, and influence adaptive immune responses [12,28]. In parallel, NLRP3 (NOD-, LRR- and pyrin domain-containing protein 3) inflammasome activation promotes the maturation of IL-1β and IL-18, further amplifying hepatic inflammation [29]. When sustained, these pathways promote hepatocellular injury, inflammatory monocyte recruitment, HSC activation, and TGF-β (transforming growth factor beta)-dependent extracellular matrix deposition, thereby linking microbial sensing to fibrosis and hepatocarcinogenesis [14]. The functional consequences of PRR activation also depend on the intensity and duration of microbial exposure. Controlled stimulation may support dendritic cell maturation, antigen presentation, and effective immune priming, whereas persistent activation, as commonly observed in cirrhosis, favors tolerogenic signaling, impaired antigen presentation, and immune exhaustion (Figure 3) [13].

Experimental evidence indicates that intestinal microbiota can promote hepatocarcinogenesis through excessive TLR4 activation in resident hepatic cells, triggering NF-κB- and STAT3-dependent survival signaling and the induction of mitogenic mediators, including epiregulin, amphiregulin, and hepatocyte growth factor. These pathways collectively enhance cellular proliferation and resistance to apoptosis [30]. In human NAFLD (non-alcoholic fatty liver disease)-related HCC, bacterial extracts derived from patients promoted regulatory T cell expansion and attenuated CD8^+^ T cell and monocyte responses ex vivo [31]. These findings indicate that HCC-associated microbiota can simultaneously activate protumorigenic innate signaling and reprogram the hepatic immune environment toward myeloid suppression and impaired cytotoxic immunity.

HCC-specific evidence indicates that microbiota-driven signals can reshape myeloid cell function within the hepatic tumor microenvironment. Intestinal dysbiosis promotes a TLR4-dependent expansion of hepatic monocytic myeloid-derived suppressor cells, accompanied by reduced CD8^+^ T cell abundance and impaired T cell proliferation [26]. Consistent with these findings, immunosuppressive myeloid-derived suppressor cell populations are expanded in patients with HCC and can suppress effector T cell responses while promoting regulatory T cell development [32]. Dendritic cell dysfunction also contributes to immune tolerance, with regulatory CD14^+^CTLA-4^+^ (cytotoxic T-lymphocyte-associated protein 4) dendritic cells suppressing T cell activity through IL-10 and IDO (indoleamine 2,3-dioxygenase)-dependent mechanisms in HCC [33]. More recently, microbiota-derived extracellular vesicles from *Klebsiella pneumoniae* have been shown to promote an M2-like macrophage phenotype, further supporting a direct link between microbial signals and protumorigenic myeloid polarization in the liver [34]. Collectively, these findings support a model in which microbiota-associated myeloid reprogramming contributes to impaired cytotoxic immunity and maintenance of an immunosuppressive HCC microenvironment.

Microbiota-derived signals may also influence innate cytotoxic populations. NK cells normally eliminate malignant cells through perforin- and granzyme-mediated cytotoxicity and support broader immune activation through the secretion of interferons, TNF-α, and granulocyte–macrophage colony-stimulating factor. Experimental depletion of commensal microbiota has been associated with impaired NK cell activity and accelerated tumor growth, although much of this evidence derives from non-HCC models [35]. Similarly, dysbiosis may reprogram innate lymphoid cells toward tumor-promoting phenotypes, further altering the balance between immune surveillance and immune escape [36].

Overall, innate microbial sensing functions as a regulatory interface between the gut microbiome and hepatic antitumor immunity. Persistent activation of PRR-dependent pathways promotes inflammatory and fibrogenic signaling, reprograms myeloid cell function, and indirectly suppresses cytotoxic lymphocyte activity. These interconnected mechanisms create a tumor-permissive immune environment and provide a biological link between dysbiosis, HCC progression, and potentially reduced responsiveness to immune checkpoint inhibition. Beyond direct recognition of microbial components, the gut microbiome also shapes hepatic immunity through the production and transformation of bioactive metabolites that can exert local and systemic immunomodulatory effects.

### 3.3. Microbial Metabolites as Immune Modulators

Microbiota-derived metabolites represent an important mechanism through which the gut microbiome regulates hepatic and systemic immunity. Short-chain fatty acids (SCFAs), particularly butyrate, support immune homeostasis through epigenetic regulation and the promotion of regulatory T cell differentiation [37]. Conversely, dysbiosis may increase the production of potentially harmful metabolites, including ethanol and ammonia, thereby contributing to hepatotoxicity, inflammation, and immune dysfunction [38]. Tryptophan-derived metabolites also modulate mucosal and hepatic immunity through activation of the aryl hydrocarbon receptor, whereas impaired signaling along this pathway has been associated with intestinal inflammation and liver injury [39].

Inosine has emerged as a relevant mediator of microbiome-dependent antitumor immunity. Produced by commensal species such as *Akkermansia muciniphila* and *Bifidobacterium pseudolongum*, inosine can enhance antigen presentation and cytotoxic T cell activity through IFN-γ (interferon)- and TNF-α-dependent mechanisms [40,41]. Signaling through the adenosine A2A receptor further promotes Th1 (T helper 1) differentiation and improves responsiveness to immune checkpoint inhibitors in preclinical models [41]. Consistent with these observations, inosine-producing bacterial taxa have been reported to be enriched among patients with HCC who respond to immunotherapy [17]. Other microbial metabolites, including anacardic acid, may also enhance antitumor immunity by activating macrophage MAPK (mitogen-activated protein kinase) signaling and promoting neutrophil extracellular trap formation, although the available evidence remains limited [16].

Bile acids constitute a particularly important interface between the intestinal microbiota and hepatic immunity. Microbial conversion of primary into secondary bile acids modifies their biological activity and enables signaling through receptors such as FXR (farnesoid X receptor) and TGR5 (Takeda G protein-coupled receptor 5), which regulate inflammatory pathways and immune cell function [42,43]. Individual bile acid derivatives may exert distinct immunological effects: 3-oxolithocholic acid suppresses Th17 differentiation through inhibition of RORγt (retinoic acid receptor-related orphan receptor gamma t), whereas isoDCA (iso-deoxycholic acid) promotes regulatory T cell differentiation by altering dendritic cell activity [44,45]. Through these pathways, the microbiota-dependent bile acid pool may influence the balance between pro-inflammatory and immunoregulatory responses within the hepatic microenvironment.

HCC-specific studies provide direct evidence that microbial metabolites influence both hepatocarcinogenesis and antitumor immunity. In obesity-associated HCC, microbiota-derived deoxycholic acid induces hepatic stellate cell senescence and a tumor-promoting senescence-associated secretory phenotype [46]. Conversely, microbiota-dependent bile acid composition regulates hepatic immune surveillance by controlling CXCL16 (C-X-C motif chemokine ligand 16) expression in liver sinusoidal endothelial cells and the recruitment of antitumor CXCR6^+^ NKT cells [47]. Short-chain fatty acids may also exert context-dependent effects: *Bifidobacterium pseudolongum*-derived acetate suppresses NAFLD-associated HCC through GPR43 (G protein-coupled receptor 43)-mediated inhibition of IL-6/JAK1 (Janus kinase 1)/STAT3 signaling, whereas patients with NAFLD-related HCC exhibit an SCFA-enriched microbial metabolomic profile associated with Treg expansion and attenuated CD8^+^ T cell responses [31]. Clinically, fecal ursodeoxycholic and ursocholic acids have been enriched among patients with unresectable HCC responding to immune checkpoint inhibition, supporting the potential value of microbial metabolites as biomarkers of therapeutic response [48]. Bile acids and SCFAs therefore represent major mediators of microbiome–immune crosstalk, influencing hepatic inflammation, immune surveillance, tumor progression, and responses to immunotherapy. These metabolic signals ultimately converge within the tumor microenvironment, where they shape the composition, activation state, and antitumor function of resident and infiltrating immune cells.

### 3.4. Tumor Microenvironment and Immune Cell Function

The HCC tumor microenvironment represents a dynamic interface in which tumor cells interact with immune, stromal, and microbiota-derived signals. Chronic hepatic inflammation and dysbiosis-associated immune modulation can reshape this ecosystem by altering antigen presentation, cytotoxic lymphocyte activity, and myeloid cell polarization [26].

Dendritic cell function appears particularly relevant to immunotherapy responsiveness in HCC, as cDC1 (conventional type 1 dendritic cell) abundance and intratumoral dendritic cell–T cell interactions have been associated with CD8^+^ T cell differentiation and clinical response to PD-1 (programmed cell death protein 1) blockade [49,50]. Microbial metabolites further shape innate lymphocyte function; acetate derived from *Lactobacillus reuteri* suppresses IL-17A-producing ILC3s (group 3 innate lymphoid cells) and enhances PD-1/PD-L1 (programmed death-ligand 1) blockade, whereas butyrate promotes CXCL11-dependent NK cell infiltration and antitumor activity in HCC [51,52]. In parallel, HCC-associated tumor signals can drive loss of cytotoxic NK-like phenotypes and promote ILC plasticity within the tumor microenvironment [53]. Bacterial extracellular vesicles may provide an additional mechanism of gut–liver immune communication, as Klebsiella pneumoniae-derived vesicles can induce immunosuppressive M2-like macrophage polarization and suppress macrophage antitumor activity [34].

Through these interconnected mechanisms, the microbiome may help shape the tumor immune landscape toward either an immune-infiltrated, therapeutically responsive state or an immune-excluded, immunosuppressive phenotype. By influencing antigen presentation, effector-cell recruitment, cytotoxic activity, innate lymphoid-cell function, and myeloid suppression, microbiota-derived signals may therefore modulate the immunological context in which immune checkpoint inhibitors exert their effects. These mechanistic observations provide the biological rationale for the growing clinical evidence linking gut microbiome composition and function with immunotherapy outcomes across solid tumors and, increasingly, in HCC.

## 4. Clinical Evidence Linking the Gut Microbiome to Immunotherapy Outcomes in Solid Tumors and HCC

### 4.1. Clinical Evidence in Solid Tumors

Clinical evidence supporting a relationship between the gut microbiome and immune checkpoint inhibitor efficacy has accumulated across multiple solid tumors, particularly melanoma [54,55,56,57,58,59] and lung cancer [60,61,62,63], with more recent studies extending these observations to renal cell carcinoma [8], urothelial carcinoma [64], and gastrointestinal malignancies [65,66,67]. Across these tumor types, numerous clinical studies have demonstrated distinct microbial profiles between responders and non-responders, while antibiotic exposure has consistently emerged as a negative predictor of immunotherapy outcomes.

Although specific microbial signatures vary considerably between studies owing to differences in patient populations, geographic location, diet, sequencing methodologies, and analytical approaches, the overall body of evidence supports a biologically relevant role of the gut microbiome in modulating antitumor immunity. Interventional studies, including fecal microbiota transplantation (FMT), further strengthen the concept that microbiome manipulation may enhance responsiveness to ICIs. Nevertheless, microbiome-based biomarkers remain investigational, and their clinical implementation is limited by the lack of standardized methodologies and prospective validation. The main clinical studies evaluating the gut microbiome in solid tumors treated with ICIs are summarized in Table 1.

Building upon this evidence, the following section focuses specifically on hepatocellular carcinoma, where the unique interplay between chronic liver disease, cirrhosis, the gut–liver axis, and antitumor immunity provides a distinct biological context for investigating microbiome-associated determinants of immunotherapy response.

### 4.2. Clinical Evidence in HCC

To date, clinical evidence evaluating the role of the gut microbiome in patients with advanced HCC remains limited, with only a small number of studies published over the past few years. The earliest study, conducted by Zheng et al. in 2019 [17], included only eight patients and established the initial framework for subsequent investigations. Since then, several studies published between 2021 and 2024 have further investigated the association between the gut microbiome and immunotherapy response in HCC [48,68,69,70,71,72,73]. Across these studies, sample sizes remain relatively small, typically ranging from fewer than 10 to approximately 90 patients, and are predominantly derived from Asian populations, with only limited representation from Western cohorts. Key characteristics and findings of these studies are summarized in Table 2.

Overall, patient populations and clinical characteristics show several shared features. Most cohorts were derived from Asian populations and were predominantly composed of patients with hepatitis B virus-related HCC, while Western data remain limited and are characterized by more heterogeneous etiologies, including hepatitis C, alcohol-related liver disease, and non-alcoholic steatohepatitis [68]. In addition, the majority of patients had preserved liver function (Child–Pugh A), reflecting typical eligibility criteria for immunotherapy studies and limiting the representation of patients with more advanced cirrhosis.

Treatment strategies also varied across studies. Although all cohorts included patients receiving ICIs, differences existed in the specific agents used, treatment lines, and the use of combination therapies, including anti-angiogenic agents [69] or locoregional approaches [70]. These differences reflect real-world clinical practice but introduce variability in the context in which microbiome–response associations are evaluated.

Methodological approaches were broadly similar but not uniform across studies. Most investigations employed prospective observational designs with baseline fecal sample collection prior to treatment initiation. However, sampling strategies varied, with some studies limited to baseline analysis [71] and others incorporating longitudinal sampling to evaluate microbiome dynamics during therapy [17,68,69,70].

Differences in microbiome analysis techniques further contribute to methodological variability. While several studies relied on 16S rRNA gene sequencing, others employed shotgun metagenomic sequencing [17,69,70], and in some cases multi-kingdom approaches including fungal profiling [69]. These differences in sequencing platforms and analytical resolution may influence the detection of microbial signatures and limit comparability across studies.

In contrast to the heterogeneity observed across clinical cohorts, preclinical studies provide more consistent mechanistic evidence supporting a role for the gut microbiome in HCC development and antitumor immunity. Intestinal microbiota can promote hepatocarcinogenesis through TLR4-dependent inflammatory signaling, while dysbiosis may reshape the hepatic immune microenvironment by expanding monocytic myeloid-derived suppressor cells and reducing antitumor T cell activity [26,30]. Microbiota-dependent bile acid metabolism can also regulate hepatic immune surveillance by modulating CXCL16 expression and the accumulation of CXCR6^+^ NKT cells [47]. Short-chain fatty acids provide an additional mechanistic link: acetate derived from *Lactobacillus reuteri* suppresses IL-17A-producing ILC3s and enhances the antitumor activity of PD-1/PD-L1 blockade, whereas butyrate promotes CXCL11-dependent NK cell infiltration and inhibits HCC growth [51,52]. These experimental findings therefore support a causal relationship between microbial activity and hepatic antitumor immunity; however, their translation into human disease has been considerably less consistent, particularly when attempting to identify specific microbial taxa or community-level signatures associated with treatment response.

Consistent with this translational gap, current studies do not demonstrate consistent microbiome signatures associated with immunotherapy response in HCC. While several investigations report differences in microbial diversity between responders and non-responders [17,48,69,70,72], these findings are not uniformly reproducible [68,71,73]. In particular, some studies observed higher microbial diversity or enrichment of specific taxa in responders [17], whereas others failed to identify significant differences in either alpha or beta diversity [71,73]. Attempts to define predictive microbial patterns, including the use of compositional ratios such as the Firmicutes-to-Bacteroidetes or Prevotella-to-Bacteroides ratios [73], have been proposed, but remain inconsistent across cohorts.

Importantly, the identification of specific taxa associated with response or resistance varies substantially between studies, with limited overlap, further underscoring the lack of reproducibility. This variability may be partly explained by multiple factors that are difficult to control, including diet, lifestyle, and host-related variables, even in studies that excluded recent antibiotic exposure [17,48,68,69,70]. Additionally, methodological differences and small sample sizes further contribute to discordant findings. Longitudinal analyses have also yielded conflicting results, with some studies reporting early microbiome changes during treatment [69], while others observed no significant temporal variation [73]. Taken together, these findings suggest that although a microbiome signal may exist, it remains inconsistent and insufficiently robust for clinical application.

Beyond commonly acknowledged limitations such as small sample sizes and the lack of validation cohorts, a major constraint of current evidence is the inability to establish a causal relationship between the gut microbiome and treatment response. Most available studies are observational and rely on baseline or longitudinal associations, which do not allow for differentiation between predictive, prognostic, or treatment-induced microbiome changes. Consequently, it remains unclear whether specific microbial profiles directly influence immunotherapy efficacy, are themselves altered by treatment, or simply reflect underlying host-related factors.

Collectively, these studies represent an important early step in understanding the potential role of the gut microbiome in modulating immunotherapy response in HCC. Although biologically plausible, given the well-established interactions between the gut microbiota and the immune system, current evidence remains limited and inconsistent. The observed variability across studies likely reflects the complex and multifactorial nature of the microbiome, which is influenced by host characteristics, disease etiology, environmental factors, and methodological differences.

At present, the microbiome cannot be considered a reliable predictive biomarker for clinical decision-making in HCC. However, its potential as both a stratification tool and a therapeutic target warrants further investigation. Future studies will require larger, well-characterized cohorts with standardized methodologies and integrated clinical, environmental, and molecular data to improve reproducibility and enable meaningful comparisons. Ultimately, a more precise understanding of microbiome–host–tumor interactions may contribute to the development of more individualized therapeutic strategies in HCC.

### 4.3. Clinical Applicability of Microbiome Biomarkers in HCC

Despite increasing evidence linking gut microbiome composition with immunotherapy outcomes, microbiome profiling is not currently suitable for routine treatment selection in HCC. Available studies are generally small, predominantly single-center, geographically concentrated, and characterized by substantial methodological heterogeneity. Moreover, no microbiome-derived classifier has undergone adequate external validation, and clinically applicable thresholds capable of prospectively distinguishing patients likely to benefit from immune checkpoint inhibitors have not been established. Importantly, there is also no evidence that microbiome-guided treatment selection improves patient outcomes. Therefore, in the near term, the microbiome is unlikely to function as a stand-alone binary biomarker for selecting or excluding patients from immunotherapy.

A more plausible clinical role would be as a complementary component of a multidimensional biomarker model. Microbiome-derived features may provide information regarding host immune status, intestinal barrier function, microbial metabolism, and environmental exposures that is not fully captured by tumor-centered biomarkers. Such information could potentially be integrated with liver function, disease etiology, tumor burden, circulating biomarkers, systemic inflammatory indices, and molecular characteristics. Microbiome profiling may also be suitable for longitudinal assessment because stool samples can be collected repeatedly during treatment. However, whether microbiome-derived data provide incremental predictive value beyond established clinical and molecular variables remains unknown and should be formally evaluated rather than assumed.

The clinical characteristics of microbiome biomarkers differ from those of other biomarkers currently investigated in HCC (Table 3). PD-L1 expression provides information directly related to the tumor immune microenvironment but require tumor tissue and may be affected by spatial and temporal heterogeneity [74]. Tumor mutational burden provides a genomic measure of potential tumor immunogenicity but has not demonstrated sufficiently consistent predictive performance to guide routine immunotherapy selection in HCC [75]. Circulating biomarkers, including circulating tumor DNA, circulating proteins and cytokines, offer minimally invasive and potentially dynamic assessment, although their clinical validation remains incomplete [76,77]. Inflammatory indices such as the neutrophil-to-lymphocyte ratio are inexpensive and readily available but are non-specific markers of systemic inflammation, with current HCC evidence supporting primarily prognostic associations rather than validated treatment-specific predictive utility [78]. In comparison, microbiome profiling is non-invasive and biologically capable of capturing host–environment interactions, but it is particularly vulnerable to pre-analytical variation, geographic and dietary influences, medication exposure, and cirrhosis-related confounding. Consequently, the microbiome cannot currently be considered superior to these other candidate biomarkers, and its value is more likely to derive from integration with, rather than replacement of, existing approaches.

## 5. Methodological and Clinical Challenges in Microbiome Biomarker Development

Despite growing interest in the gut microbiome as a potential predictor of immunotherapy response across solid tumors, the current body of evidence remains methodologically heterogeneous and often difficult to interpret [79]. Most available clinical studies are limited by small sample sizes and exploratory designs, reducing the reproducibility and generalizability of reported findings [17]. Consequently, results have remained inconsistent, with some studies identifying significant associations between microbiome composition and therapeutic response [72], whereas others have failed to confirm these relationships [73].

This lack of reproducibility raises important methodological and analytical questions. For a biomarker to be clinically applicable, findings must be robust and consistent across independent cohorts, a criterion that current microbiome studies have not yet fulfilled. Nevertheless, these inconsistencies should not be interpreted as evidence against a microbiome–immunotherapy interaction. Rather, they likely reflect substantial heterogeneity in study design, patient populations, sampling strategies, and analytical methodologies. Understanding these sources of variability is essential for interpreting existing data and for guiding the development of more standardized and reproducible approaches in future research.

### 5.1. Pre-Analytical Variability

Pre-analytical variability represents an important but often underappreciated source of heterogeneity in microbiome studies. Among these factors, stool collection methods are rarely standardized or consistently reported. In many studies, the procedures used for sample collection are not described in detail, while only a minority explicitly reference standardized protocols, such as the International Human Microbiome Standards (IHMSs) guidelines or the Human Microbiome Project (HMP) collection kits [17,69,70].

Storage conditions represent another critical source of pre-analytical variability in microbiome studies. Most studies report that stool samples were stored at −80 °C following collection; however, the time interval between sample collection and freezing is not consistently standardized or clearly reported. In several cases, samples were frozen within 24–48 h [48,73], but delays prior to freezing may allow for ongoing microbial metabolic activity and shifts in community composition.

The timing of stool collection also varies considerably across studies and represents an additional source of methodological heterogeneity. While all studies include baseline sampling prior to treatment initiation, subsequent sampling strategies differ substantially. Some studies perform only limited follow-up sampling, typically at progression or predefined time points [69,72,73], whereas others incorporate longitudinal sampling throughout treatment, either at each cycle or at regular intervals. Notably, at least one study relied exclusively on baseline samples without any longitudinal assessment [71].

Collectively, variability in pre-analytical factors, including stool collection procedures, pre-freezing handling, and sampling strategies, represents a significant source of bias across studies. Differences in sample collection conditions, delays prior to freezing, and reliance on either single-time-point or longitudinal sampling may all influence the observed microbiome composition and its temporal dynamics. These inconsistencies in sample handling and study design limit comparability across cohorts and may contribute to the heterogeneity of reported findings.

### 5.2. Analytical Variability

DNA extraction and sequencing methodologies represent a major source of analytical variability across microbiome studies. Substantial heterogeneity exists in the choice of DNA extraction kits, library preparation protocols, sequencing platforms, and downstream bioinformatic pipelines. While some studies employed standardized commercial kits such as QIAamp [17] or PowerFecal [73] systems, others used alternative extraction methods, such as the BIOER fecal DNA purification kit [69], each with different efficiencies in recovering microbial DNA, particularly from taxa with resistant cell walls. Consequently, differences in extraction efficiency, sequencing depth, and analytical workflows may lead to divergent results even when analyzing similar patient populations.

Similarly, sequencing approaches varied considerably, with most studies relying on 16S rRNA gene sequencing targeting the V3–V4 regions, while others utilized whole-genome shotgun metagenomic sequencing [17,69,70,73]. These techniques are not directly comparable, as 16S sequencing provides limited taxonomic resolution, typically at the genus level, and does not capture functional microbial capacity. In contrast, shotgun metagenomics enables species-level identification and allows for functional and pathway-level analyses [80]. These methodological differences have important implications for data interpretation.

The assessment of microbial diversity represents a central component of microbiome studies and another major source of analytical variability; however, the selection and interpretation of diversity metrics are not standardized across studies. Most investigations evaluate alpha diversity using indices such as Shannon [48,69,70,71,72,73], Simpson [70], or Chao1 [68,70,71,73] which capture different aspects of microbial richness and evenness, while beta diversity is commonly assessed using Bray–Curtis dissimilarity and visualized through principal coordinate analysis.

Although these approaches are widely used, they are not directly interchangeable, and differences in metric selection may influence study conclusions. For example, richness-based indices such as Chao1 may yield different results compared to diversity measures that account for both richness and evenness, such as the Shannon index. Similarly, beta diversity analyses often rely on visual clustering, which may be subject to interpretation and dependent on the statistical methods applied [80]. As a result, variability in the choice of diversity metrics and analytical approaches may contribute to inconsistent findings across studies. Importantly, differences in microbial diversity do not necessarily reflect functionally relevant changes, further complicating the interpretation of microbiome–treatment associations.

### 5.3. Clinical Variability

Clinical heterogeneity represents a particularly important challenge in microbiome studies of HCC because several factors that influence treatment outcomes also independently shape the gut microbial ecosystem. These include treatment regimen and line of therapy, the severity and ethology of the underlying liver disease, concomitant medications, nutritional status, and geographic and dietary exposures. Inadequate control of these variables may therefore confound apparent associations between microbiome composition and immunotherapy efficacy.

Although all available studies evaluated patients receiving ICIs, treatment strategies were not uniform. Some cohorts included combinations with tyrosine kinase inhibitors [70,71] or locoregional treatments such as transarterial chemoembolization [70], whereas others evaluated ICI monotherapy. Moreover, different ICIs were used across studies, including nivolumab, pembrolizumab, atezolizumab, and camrelizumab, reflecting regional practice patterns and the evolution of systemic treatment strategies. Treatment line represents an additional source of heterogeneity, as some cohorts included patients treated in the first-line setting, whereas others enrolled patients previously exposed to systemic therapies. Such populations are not directly comparable, since prior treatment and more advanced disease may influence clinical condition, nutritional intake, and potentially microbiome composition.

The severity of underlying chronic liver disease represents a particularly important source of heterogeneity in HCC microbiome studies. Cirrhosis itself profoundly alters gut microbiome composition, yet its presence and severity have been inconsistently characterized across available cohorts [81]. Cirrhosis was reported in approximately half of the patients in some studies [70,73], whereas other cohorts included exclusively cirrhotic patients [68] or did not clearly document cirrhosis status. This distinction is particularly relevant in HBV-associated HCC, which may arise in the absence of established cirrhosis [81].

Liver functional reserve was more consistently documented, although the populations remained heterogeneous: some cohorts were restricted to Child–Pugh A patients [17,68], whereas others included varying proportions of Child–Pugh B disease [48,72,73]. Importantly, poorer Child–Pugh status was significantly more frequent among non-responders in at least one cohort [71], illustrating how hepatic dysfunction itself may be associated with treatment outcome and potentially confound microbiome–response relationships. Portal hypertension was even less consistently characterized.

Endoscopic evidence of portal hypertension or varices was documented in only a limited number of studies, while most cohorts did not systematically report this variable [68,71]. Because Child–Pugh class, portal hypertension, and hepatic decompensation may influence intestinal permeability, bacterial translocation, bile acid metabolism, systemic inflammation, and microbial composition [5], microbial signatures associated with poor immunotherapy outcomes may partly reflect more advanced liver disease rather than treatment-specific resistance. Future studies should therefore characterize cirrhosis status and severity in greater detail and incorporate these variables into stratified or multivariable analyses rather than treating cirrhosis as a simple binary characteristic.

HCC ethology introduces an additional layer of biological heterogeneity, as viral hepatitis, alcohol-related liver disease, and metabolic dysfunction-associated steatotic liver disease may be associated with distinct microbial and immunological profiles [82]. The etiologic distribution differed markedly across the available studies. Several Asian cohorts were predominantly or almost exclusively HBV-associated [48,73], whereas others included mixtures of HBV, HCV, alcohol-related, and non-B/non-C disease. In contrast, the Western cohort included a broader representation of HBV-, HCV-, NAFLD-, and alcohol-related cirrhosis [68]. These differences are relevant because microbial signatures identified predominantly in HBV-associated Asian populations may not be directly generalizable to cohorts enriched for alcohol-related or metabolic liver disease [82]. Ethology should therefore be considered not merely as a baseline demographic variable, but as a potential biological confounder when comparing microbiome signatures across studies.

Concomitant medication exposure was also inconsistently controlled. Antibiotics, among the strongest modifiers of gut microbial composition and diversity, were addressed using substantially different approaches across HCC cohorts [82]. Some studies excluded antibiotic exposure during defined pretreatment periods ranging from 1 to 3 months [48,68,69,70], whereas others recorded recent exposure [71,73] or only reported that antibiotics were not administered during treatment. Recent antibiotic exposure was associated with absence of disease control in one cohort, further illustrating its potential to confound microbiome–outcome associations. PPI (proton pump inhibitors) exposure was even less uniformly managed, being excluded in some studies, recorded in others, and not reported in several cohorts [17,69,72].

Cirrhosis-directed therapies represent an additional and largely neglected source of confounding. Lactulose and rifaximin, commonly used for the prevention or treatment of hepatic encephalopathy, may modify intestinal microbial composition or function [82], yet neither was systematically documented as an individual exposure in the available HCC microbiome studies. Nonabsorbable disaccharides and recent antibiotic exposure were indirectly controlled in one cohort [68], but lactulose and rifaximin were not specifically analyzed. These medications may also serve as markers of more advanced liver disease, further complicating efforts to distinguish treatment-specific microbial signatures from those associated with hepatic decompensation.

Nutritional and environmental factors were among the least consistently assessed variables. Diet, lifestyle, comorbidities, and geographic location are established determinants of gut microbiome composition [82]. Formal nutritional assessment was absent from nearly all available HCC cohorts. BMI was reported in only a limited number of studies [68,71], while weight loss, sarcopenia, nutritional scores, and dietary intake were generally not evaluated. In patients with HCC and cirrhosis, malnutrition, reduced oral intake, and sarcopenia may introduce additional variation in both dietary exposure and microbial metabolism [83].

Dietary characterization was similarly limited. One cohort attempted to reduce extreme dietary variation by excluding unusual eating patterns and recent consumption of yogurt, probiotics, or prebiotics, but formal dietary assessment was not performed [70]. Most other studies did not systematically evaluate dietary intake. This limitation is particularly relevant given the geographic concentration of current evidence, with most cohorts originating from China, Taiwan, South Korea, or Japan and only limited Western representation. Regional dietary patterns may therefore contribute to differences in baseline microbiome composition and partly explain the limited reproducibility of microbial signatures across populations [84]. Future studies should systematically document nutritional status and dietary exposures and validate candidate microbiome biomarkers across geographically and etiologically diverse cohorts.

Taken together, HCC presents an unusually complex setting for microbiome biomarker development because liver disease severity, ethology, treatment exposure, concomitant medications, and nutritional and environmental factors may simultaneously affect both microbiome composition and clinical outcomes. Failure to account for these variables may result in microbial profiles that appear predictive of ICI response but instead reflect underlying liver dysfunction, prognosis, or population-specific exposures. Accordingly, methodological harmonization alone will not be sufficient. Future studies should incorporate detailed clinical phenotyping and demonstrate that predefined microbiome-derived signatures are analytically reproducible, externally validated across etiologically and geographically diverse populations, and capable of providing predictive information beyond established clinical, inflammatory, circulating, and tumor-based biomarkers.

### 5.4. Strengths and Limitations of Current HCC Evidence

Current HCC studies support an association between the gut microbiome and immunotherapy outcomes, but no reproducible microbial signature has yet emerged. Most cohorts are small and exploratory, limiting statistical power and increasing the likelihood of cohort-specific associations. Independent external validation remains limited, and methodological differences further reduce comparability. In particular, 16S rRNA sequencing, shotgun metagenomics, and multi-kingdom approaches differ substantially in taxonomic and functional resolution, meaning that apparent disagreement between studies may partly reflect analytical differences rather than true biological inconsistency.

Clinical heterogeneity adds another major source of variability. Studies have used different definitions of treatment benefit, including objective response, disease control, and durable clinical benefit, and have evaluated ICI monotherapy as well as combinations with anti-angiogenic agents, tyrosine kinase inhibitors, or locoregional therapies. In addition, HCC-specific confounders such as cirrhosis severity, ethology, medication exposure, nutritional status, geography, and diet are inconsistently controlled. Together, small sample sizes, limited external validation, heterogeneous sequencing platforms, variable response definitions, treatment heterogeneity, and incomplete adjustment for clinical confounders likely explain much of the poor reproducibility across studies. Until these issues are addressed in prospective multicenter cohorts using standardized methodologies, microbiome signatures should remain considered exploratory rather than clinically validated predictive biomarkers.

## 6. Therapeutic Manipulation of the Microbiome in HCC

**Figure 4:**
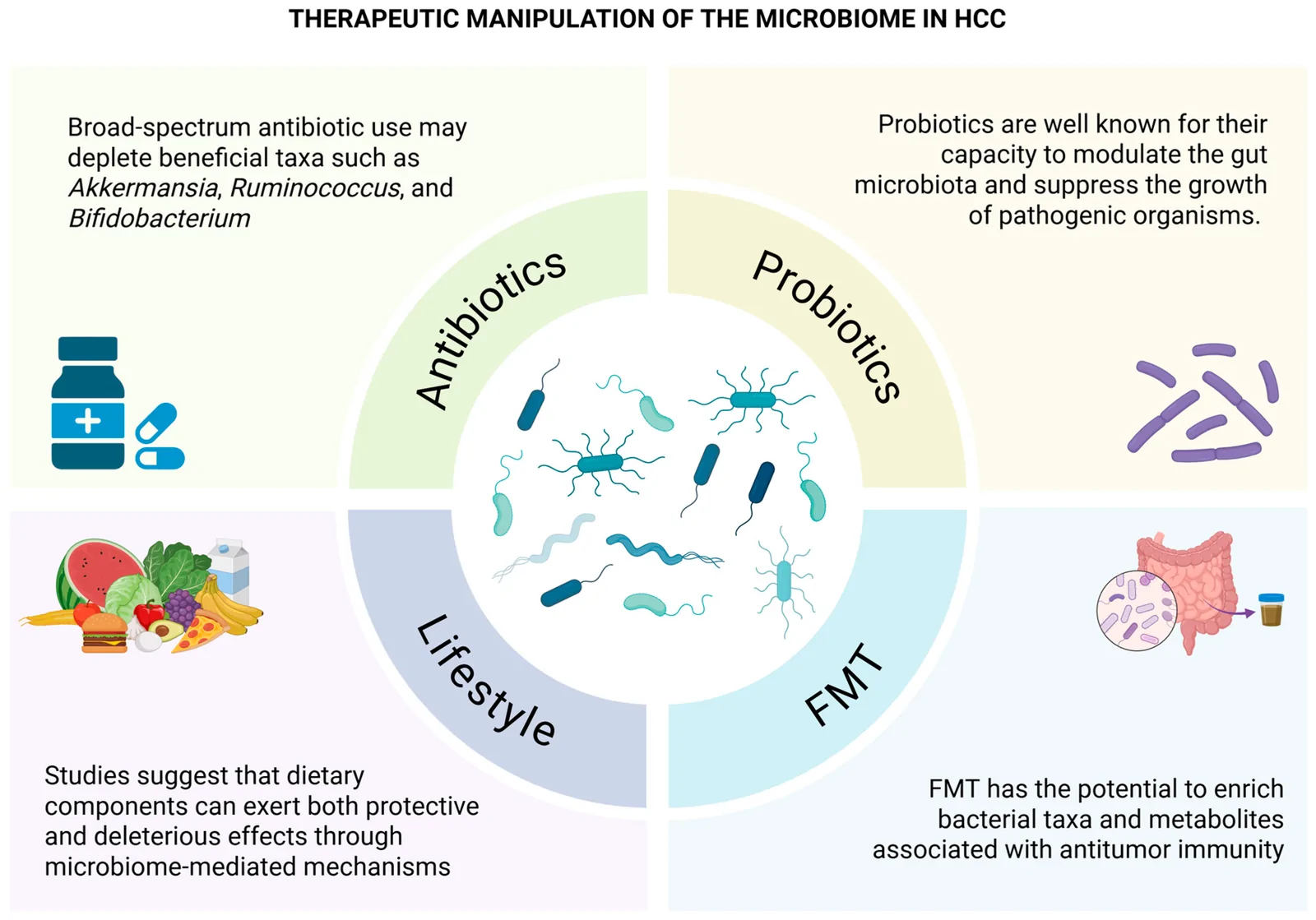
Therapeutic manipulation of the microbiome in HCC. Multiple strategies have been proposed to modulate the microbiome in HCC, including antibiotics, probiotics, dietary and lifestyle interventions, and FMT. Broad-spectrum antibiotics may disrupt beneficial bacterial taxa associated with antitumor immunity, whereas probiotics and FMT may promote enrichment of favorable microbial communities and metabolites. Dietary and lifestyle factors can further influence microbiome composition and immune regulation, providing a rationale for the development of microbiome-targeted therapeutic strategies in HCC. Created in BioRender. Ostafe, M. (2026). https://BioRender.com/deus6ho.

The gut microbiota plays a central role in host physiology, contributing to nutrient metabolism, energy homeostasis, and the regulation of immune responses [81]. Disruption of this ecosystem, termed dysbiosis, is characterized by alterations in microbial composition and function and has been implicated in the pathogenesis and progression of multiple diseases [85]. In HCC, particularly in the context of cirrhosis, studies have demonstrated a distinct dysbiotic profile [86], suggesting a potential role for the microbiome in shaping disease biology and therapeutic outcomes. Building on emerging evidence that microbiome composition may influence response to systemic therapies—especially ICIs —the gut microbiota has been proposed as a modifiable determinant of treatment efficacy, representing a promising target for therapeutic intervention (Figure 4).

### 6.1. Antibiotics

Antibiotics represent one of the most potent disruptors of the gut microbiome and have been widely investigated as modifiers of cancer therapy outcomes. In preclinical models, their effects appear context-dependent. For instance, in murine models, antibiotic-induced depletion of commensal bacteria has been associated with reduced tumor burden, potentially through alterations in bile acid metabolism leading to enhanced hepatic NKT cell-mediated immunosurveillance [47]. Similarly, selective microbiota depletion with vancomycin was shown to suppress hepatocarcinogenesis, likely by eliminating bacterial taxa and metabolites involved in tumor initiation and progression [87].

In contrast, clinical data consistently suggest a detrimental association. Retrospective analyses have reported that antibiotic exposure is associated with worse survival in patients with HCC treated with sorafenib [88] and similar findings have been observed in large pooled analyses of patients receiving ICIs [89]. Mechanistically, broad-spectrum antibiotic use may deplete beneficial taxa such as *Akkermansia*, *Ruminococcus*, and *Bifidobacterium*, while promoting expansion of immunosuppressive microbial communities, thereby impairing antitumor immunity. However, given the frequent need for antibiotic therapy in cirrhotic patients, causality remains difficult to establish.

### 6.2. Probiotics

Probiotics, defined as live microorganisms that confer health benefits to the host, are well known for their capacity to modulate the gut microbiota and suppress the growth of pathogenic organisms [90]. Moreover, they can interact with the gastrointestinal mucosa, thereby influencing systemic immune responses. In this context, probiotics have been proposed as potential modulators of tumor response to immunotherapy, and even as therapeutic agents in their own right [91]. However, evidence remains limited, particularly in HCC.

In a murine study, a probiotic mixture consisting of *Lactobacillus rhamnosus*, *Escherichia coli Nissle 1917*, and heat-inactivated VSL#3 (multi-strain probiotic formulation) was evaluated for its effects on hepatocellular tumor growth, angiogenesis, and Th17 cell modulation. The authors reported a reduction in liver tumor growth of approximately 40%, associated with inhibition of angiogenesis, particularly IL-17-dependent pathways, and modulation of the TME. Additionally, probiotic administration altered the structural and functional composition of the gut microbiota, increasing anti-inflammatory bacteria and metabolites in the intestine [90]. In humans, however, no prospective studies have demonstrated an effect of probiotic supplementation on treatment response in HCC.

These findings highlight the biological plausibility of probiotic-based modulation of antitumor immunity; however, the lack of clinical evidence and the reliance on preclinical models limit their immediate translational applicability, and their clinical utility in HCC remains uncertain.

### 6.3. Fecal Microbiota Transplantation

FMT has emerged as a potential microbiome-directed strategy in HCC based on its capacity to modulate gut microbial composition and function, thereby influencing hepatic inflammation and immune homeostasis [92]. In this context, FMT may enhance responsiveness to ICIs, particularly given evidence linking antibiotic-induced microbiome disruption to poorer immunotherapy outcomes [8]. By restoring a favorable microbial ecosystem, FMT has the potential to enrich bacterial taxa and metabolites associated with antitumor immunity. Although FMT has not yet been specifically evaluated in HCC cohorts, data from other malignancies are encouraging. In melanoma, transfer of fecal microbiota from responders to anti-PD-1 therapy into non-responders restored treatment sensitivity and enhanced antitumor immune responses, including increased antigen-presenting cell activation and tumor-infiltrating lymphocytes [59].

However, the application of FMT in HCC must be considered within the unique context of cirrhosis, a condition characterized by profound dysbiosis, immune dysfunction, and increased susceptibility to infection. Clinical studies in cirrhotic patients suggest that FMT can modulate microbiota composition and disease-related pathways; for example, improvements in microbiota diversity and clinical parameters have been reported following FMT administration [92,93]. Nevertheless, these findings should be interpreted with caution. Patients with cirrhosis are at increased risk of bacterial translocation and sepsis, raising significant safety concerns regarding the administration of live microbial communities. Although FMT has generally been well tolerated in selected populations, the risk of infectious complications, the heterogeneity of protocols, and the lack of standardized donor screening strategies remain important limitations.

Emerging preclinical evidence further supports a mechanistic link between microbiome composition and hepatocarcinogenesis. In murine models, fecal microbiota derived from patients with HCC promoted liver inflammation, fibrosis, and hepatocyte proliferation, with enrichment of pro-inflammatory immune populations, including TH1 and TH17 cells, and increased abundance of Klebsiella pneumoniae [25]. These findings suggest that microbiome composition may actively contribute to tumor-promoting processes, raising the possibility that targeted microbiome modulation could have therapeutic benefit. However, such hypotheses remain speculative and require validation in human studies. In parallel, early-phase clinical efforts are exploring FMT as a strategy to overcome resistance to systemic therapies, including atezolizumab plus bevacizumab, although results are not yet available [94,95].

Taken together, while FMT represents a promising approach to modulate the gut–liver–immune axis, its application in HCC remains investigational. To date, no prospective clinical trials have demonstrated its efficacy as an adjunct to systemic therapy in HCC, and careful consideration of safety, patient selection, and clinical context is essential.

### 6.4. Diet and Lifestyle

Although not traditionally considered therapeutic interventions, diet and lifestyle represent major determinants of gut microbiome composition and may influence hepatocarcinogenesis [96]. Preclinical studies suggest that dietary components can exert both protective and deleterious effects through microbiome-mediated mechanisms. For instance, prolonged consumption of fermentable fibers such as inulin has been shown to promote liver injury, hyperbilirubinemia, and HCC development in murine models, potentially through dysbiosis-driven production of SCFAs [87].

Similarly, high dietary cholesterol has been linked to progressive liver disease and HCC development, accompanied by characteristic microbiome alterations, including enrichment of pro-inflammatory taxa and depletion of beneficial commensals [97]. Lifestyle interventions such as intermittent fasting have also demonstrated protective effects in murine models, with reduced incidence of neoplastic lesions despite comparable caloric intake [98]. In addition, host–microbiome interactions appear to be transmissible and modifiable: co-housing experiments have shown that normalization of gut microbiota can reduce HCC risk, while the transfer of obesity-associated microbiota promotes hepatocarcinogenesis in recipient mice [99,100].

Finally, epidemiological and experimental data suggest that coffee consumption may exert hepatoprotective effects, partly through modulation of the gut microbiome and anti-inflammatory pathways [101]. However, these findings are largely derived from preclinical and observational studies, and their direct applicability to patients with HCC remains to be established. This underscores the context-dependent effects of microbiome modulation, whereby dietary interventions may exert divergent effects depending on the underlying hepatic and metabolic environment.

### 6.5. Emerging Precision Approaches

Emerging strategies in microbiome modulation are increasingly focused on precision-based approaches aimed at overcoming the limitations of non-specific interventions such as FMT. Defined microbial consortia, composed of selected bacterial strains with known immunomodulatory properties, are being developed to provide more reproducible and controllable therapeutic effects [102]. In parallel, the gut microbiome is being explored as a predictive biomarker to guide patient selection, enabling identification of individuals more likely to benefit from ICIs [54]. Additionally, advances in synthetic biology have enabled the development of genetically engineered bacteria capable of delivering immunomodulatory molecules or modulating metabolic pathways, although these approaches remain in early stages of investigation [103]. Importantly, these strategies are being designed for integration with systemic therapies, including ICIs and anti-angiogenic agents, with the aim of enhancing treatment efficacy and overcoming resistance [59,96]. Collectively, precision microbiome modulation represents a promising frontier in personalized therapy for HCC, although further clinical validation is required.

In summary, therapeutic manipulation of the gut microbiome represents a compelling and biologically plausible strategy to enhance treatment efficacy in HCC. While approaches such as antibiotics, probiotics, FMT, and dietary modulation have demonstrated the capacity to influence tumor–immune interactions, current evidence remains largely preclinical or indirect, with limited validation in HCC-specific clinical settings. Importantly, the unique context of cirrhosis introduces additional complexity, particularly with regard to safety, microbial translocation, and host–microbiome interactions. Emerging precision-based strategies, including defined microbial consortia and microbiome-informed patient selection, offer the potential to overcome the limitations of non-specific interventions and move toward more personalized therapeutic paradigms. However, rigorous prospective studies, standardized methodologies, and integration of microbiome profiling into clinical trial design are essential to translate these approaches into routine clinical practice.

## 7. Future Directions: A Roadmap for Microbiome-Integrated Immunotherapy

Despite substantial advances in understanding microbiome–immune interactions in HCC, translation into clinically actionable strategies remains at an early stage. Current evidence indicates that microbiome composition and function may influence therapeutic responsiveness through modulation of hepatic inflammation, immune tolerance, and antitumor immunity along the gut–liver axis [8,10,47,54]. Consequently, microbiome-directed interventions—including probiotics, dietary modulation, and FMT—have emerged as potential approaches to enhance immunotherapy efficacy and overcome resistance in HCC, although their clinical application still requires prospective validation and methodological standardization [58,59,87,90].

Recent evidence strengthens the biological rationale for investigating defined probiotics and live biotherapeutic products as adjuncts to immunotherapy. Importantly, emerging HCC-specific data provide mechanistic support for this approach. *Bifidobacterium* supplementation enhanced anti-PD-1 activity in experimental HCC, an effect linked to the microbial metabolite isobutyrate, increased CD8^+^ T cell activation and IFN-γ production, and suppression of JAK/STAT3 signaling [104]. Similarly, *Lactobacillus kefiranofaciens*, a strain isolated from kefir grains, enhanced antitumor immunity and the efficacy of PD-1/PD-L1 blockade in HCC models through upregulation of SOCS3, inhibition of JAK–STAT signaling, reduced PD-L1 expression, and increased T and NK cell infiltration [105]. These findings are complemented by early clinical evidence from other tumor types. In a randomized phase I study in metastatic renal cell carcinoma, addition of the live bacterial product *Clostridium butyricum* MIYAIRI 588 (CBM588) to cabozantinib plus nivolumab was associated with a higher objective response rate, although the prespecified microbiome endpoint was not met [106]. Such findings are particularly relevant given the increasing commercial availability and promotion of probiotic supplements, prebiotics, and fermented foods such as yogurt. However, commercially available products should not be considered equivalent to strain-defined live biotherapeutics, as their microbial composition, viability, dose, and functional effects may differ substantially. Accordingly, current evidence does not support routine probiotic, prebiotic, or fermented food supplementation specifically to enhance ICI efficacy in patients with HCC. Future prospective trials should evaluate defined microbial strains, microbial metabolites or postbiotics, and controlled dietary interventions, while integrating longitudinal metagenomic, metabolomic, and immune profiling to determine whether their effects are reproducible across different HCC etiologies and treatment regimens.

A major priority for future research would also be the prospective validation of candidate microbiome biomarkers in adequately powered, multicenter cohorts. Such studies should include geographically and etiologically diverse populations and incorporate predefined sampling protocols, clinical endpoints, and analytical pipelines. Independent external validation will be essential to determine whether microbial signatures identified in individual cohorts are reproducible and provide predictive information beyond established clinical and molecular biomarkers [80].

Recent advances have shifted microbiome research from descriptive profiling toward mechanistic and functional characterization [80]. In this context, integrating microbiome sequencing with metabolomic approaches is essential to identify biologically relevant microbial signatures and pathways associated with HCC risk, progression, and treatment response [24,107]. Moving beyond taxonomic composition toward functional outputs—including microbial metabolic pathways, metabolite production, and host–microbiome interactions—will be critical for developing clinically actionable biomarkers and refining patient stratification strategies.

A major challenge for the field remains the lack of standardization in microbiome research methodologies. Variability in sample collection, processing, and analytical pipelines contributes to inconsistent findings across studies. Standardized and scalable approaches, including stabilized stool collection methods such as DESS (dimethyl sulfoxide + ethylenediaminetetraacetic acid + saturated NaCl)-based sampling, are essential to ensure reproducibility and enable large-scale, longitudinal investigations [80,108,109,110]. Harmonization of collection timing, storage conditions, DNA extraction, sequencing platforms, and bioinformatic workflows will therefore be crucial for translating microbiome research into clinical practice.

Looking forward, HCC should increasingly be viewed as a dynamic, multi-layered ecosystem shaped by complex interactions between the microbiome, host metabolism, immune regulation, and tumor biology. Integrating multi-omics approaches—including microbiomics, metabolomics, transcriptomics, and immunophenotyping—may significantly improve biomarker discovery, refine prognostic models, and guide therapeutic decision-making [24,107]. In parallel, advances in artificial intelligence and machine learning are creating new opportunities to integrate high-dimensional microbiome and multi-omics datasets, enabling the identification of clinically relevant microbial signatures and predictive response models that may not be detectable through conventional analytical approaches [48,80]. Future models should ideally be trained in large multicenter datasets and subsequently validated in independent cohorts to minimize overfitting and improve clinical generalizability. Together, these systems-level strategies may facilitate the development of personalized therapeutic approaches combining microbiome modulation with targeted metabolic and immunotherapeutic interventions.

Advancing microbiome-integrated oncology will also require study designs capable of capturing the dynamic nature of microbial ecosystems. Longitudinal sampling before treatment and at predefined time points during therapy may help distinguish baseline predictive signatures from treatment-induced microbiome changes and identify early microbial or functional changes associated with therapeutic response [80]. Such analyses should incorporate temporal variability, environmental influences, medication exposure, and changes in liver function to improve the interpretation and reproducibility of microbiome–treatment associations. Incorporating computational and AI-driven analytical frameworks into these longitudinal models may further enhance the ability to identify clinically actionable patterns and optimize patient stratification.

Importantly, future studies should increasingly prioritize functional rather than exclusively taxonomic characterization of the microbiome. Taxonomic differences between responders and non-responders may vary considerably between populations, whereas microbial metabolic pathways and biologically active products may represent more reproducible features of host–microbiome interactions [24,80,107]. Combining metagenomic sequencing with metabolomics and host immune profiling may therefore provide a more mechanistically informative framework for identifying biomarkers that are transferable across populations and treatment settings.

Overall, integrating microbiome-derived insights into clinical oncology holds considerable potential to refine patient selection, enhance therapeutic efficacy, and advance immunotherapy in HCC toward a more precise, personalized, and biologically informed therapeutic paradigm. Achieving this goal will require prospective multicenter validation, standardized sampling and analytical workflows, longitudinal monitoring, integration of functional multi-omics data, and rigorous validation of AI-derived predictive models before microbiome-based biomarkers can be incorporated into routine clinical decision-making.

## 8. Conclusions

The gut microbiome has emerged as a critical regulator within the complex biological landscape of HCC, linking the gut–liver axis with immune function, metabolic signaling, and the tumor microenvironment. Clinical studies consistently demonstrate that gut microbial composition is associated with response to immune checkpoint inhibitors, although no universal microbial signature has yet been identified. Among the taxa most frequently associated with favorable outcomes, *Akkermansia muciniphila*, *Faecalibacterium*, *Bifidobacterium*, and *Alistipes* have shown positive associations with treatment response or survival across different cohorts, whereas antibiotic-induced dysbiosis has consistently been linked to inferior clinical outcomes. These observations suggest that both microbial composition and microbial-derived metabolites, including short-chain fatty acids and bile acid derivatives, contribute to shaping antitumor immunity.

From a clinical perspective, microbiome profiling could complement established biomarkers, such as PD-L1 expression and clinical characteristics, to improve patient selection for immunotherapy and identify individuals less likely to benefit from immune checkpoint blockade. Beyond its role as a predictive biomarker, the microbiome also represents a potentially modifiable therapeutic target. Strategies including responsible antibiotic use, dietary interventions, probiotic or next-generation live biotherapeutic approaches, and fecal microbiota transplantation may ultimately enhance immunotherapy efficacy by restoring a more favorable microbial ecosystem, although these approaches remain investigational and require prospective validation.

Despite these promising findings, several challenges continue to limit clinical implementation. Considerable heterogeneity in sequencing methodologies, bioinformatic pipelines, patient populations, and geographic and dietary factors has resulted in inconsistent microbial signatures across studies. Furthermore, most available evidence is derived from small, predominantly retrospective cohorts, highlighting the need for large, multicenter prospective studies with standardized sampling and analytical protocols.

As our understanding of host–microbiome interactions continues to evolve, integrating microbial taxa, functional microbial pathways, and metabolomic signatures into predictive models may enable more precise patient stratification and support microbiome-guided therapeutic interventions. In HCC, where chronic liver disease, cirrhosis, and the gut–liver axis are intrinsically linked, the microbiome represents one of the most promising avenues for advancing precision immuno-oncology.

## Figures and Tables

**Table 1:** Clinical studies evaluating the association between the gut microbiome and immune checkpoint inhibitor efficacy across solid tumors.

Study	Tumor Type	Main Findings	Key Taxa
Routy et al., 2018 [8]	NSCLC, RCC	A. muciniphila was associated with improved response to PD-1/PD-L1 blockade, and responder-derived FMT restored sensitivity in mice.	*Akkermansia muciniphila*, *Ruminococcaceae*, *Alistipes indistinctus*
Gopalakrishnan et al., 2018 [54]	Melanoma	Higher gut microbial diversity and enrichment of Ruminococcaceae and Faecalibacterium were associated with improved response to anti-PD-1 therapy and successful FMT transfer in mice.	*Faecalibacterium* (*F. prausnitzii*), *Ruminococcaceae*, *Clostridiales*
Chaput et al., 2017 [55]	Metastatic melanoma	Baseline gut microbiota enriched in Firmicutes was associated with improved response to ipilimumab and increased risk of immune-mediated colitis.	*Faecalibacterium*, *Firmicutes*, *Gemmiger*, *Bacteroides*.
Frankel et al., 2017 [56]	Metastatic melanoma	Distinct gut microbiota and fecal metabolites were associated with improved response to immune checkpoint inhibitors.	*Bacteroides caccae*, *Faecalibacterium prausnitzii*, *Bacteroides thetaiotaomicron*, *Holdemania filiformis*, *Dorea formicigenerans*.
Lee et al., 2022 [57]	Advanced melanoma	Cross-cohort analysis confirmed that the gut microbiome is associated with response to immune checkpoint inhibitors, but microbial signatures showed substantial cohort-dependent variability, with no universal predictive biomarker identified.	*Bifidobacterium pseudocatenulatum, Roseburia* spp., *Akkermansia muciniphila*, *Dorea formicigenerans*, *Lactobacillus vaginalis*, *Phascolarctobacterium succinatutens* (responders); *Bacteroides clarus*, *Ruminococcus gnavus* (non-responders)
Baruch et al., 2021 [58]	Metastatic melanoma	Fecal microbiota transplantation (FMT) combined with anti-PD-1 re-induction induced objective responses in a subset of patients with PD-1-refractory melanoma and enhanced intratumoral immune activation.	*Veillonellaceae*, *Lachnospiraceae*, *Ruminococcaceae*, *Bifidobacterium adolescentis*, *Ruminococcus bromii*.
Davar et al., 2021 [59]	Metastatic PD-1-refractory melanoma	Responder-derived FMT plus pembrolizumab restored clinical benefit in PD-1-refractory melanoma and was associated with donor microbiota engraftment and enhanced antitumor immunity.	*Faecalibacterium prausnitzii*, *Bifidobacterium longum*, *Collinsella aerofaciens*, *Ruminococcaceae*, *Lachnospiraceae*, *Akkermansia muciniphila;* non-responders remained enriched for *Bacteroides* spp.
Jin et al., 2019 [60]	Advanced NSCLC (Chinese cohort, nivolumab, *n* = 37)	Higher baseline gut microbial diversity was associated with improved response to PD-1 blockade and enhanced systemic antitumor immunity.	Enriched in responders: *Bifidobacterium longum*, *Prevotella copri*, *Alistipes putredinis*, *Lachnobacterium*, *Lachnospiraceae* spp., *Shigella* spp. Enriched in non-responders: *Ruminococcus*
Derosa et al., 2022 [61]	Advanced NSCLC	Baseline Akkermansia muciniphila was associated with improved response and survival after PD-1/PD-L1 blockade.	*Akkermansia muciniphila*, *Eubacterium hallii*, *Bifidobacterium adolescentis*, *Ruminococcus* spp., *Roseburia* spp., *Alistipes* spp.
Sun et al., 2024 [62]	Extensive-stage small cell lung cancer (ES-SCLC)	Gut microbiota composition and metabolite profiles were associated with response to anti-PD-L1 immunotherapy. Responders showed higher post-treatment microbial diversity, enrichment of SCFA-producing bacteria, and increased SCFA metabolites.	*Faecalibacterium*, *Subdoligranulum*, *Clostridium sensu stricto 1*, *Dorea*, *Romboutsia*, *Coprococcus*, *Phascolarctobacterium*, *Hungatella*, *Ruminococcus* spp. (responders); *Dubosiella*, *Coriobacteriaceae* (non-responders).
Hakozaki et al., 2020 [63]	Advanced NSCLC	Higher baseline gut microbial diversity and enrichment of specific commensal bacteria were associated with improved response and survival following anti-PD-1/PD-L1 therapy. Antibiotic exposure was associated with reduced microbial diversity and poorer microbiome profiles.	*Ruminococcaceae*, *Agathobacter*, *Clostridiales* (responders); *Hungatella* (antibiotic-exposed patients); *Lactobacillaceae*, *Raoultella* (associated with less severe irAEs).
Matsumoto et al., 2025 [64]	Urothelial carcinoma	Lower oral and gut abundance of Veillonellaceae and Prevotellaceae was associated with longer overall survival and progression-free survival following immune checkpoint inhibitor therapy.	*Veillonellaceae*, *Prevotellaceae*.
Zhu et al., 2024 [65]	Biliary tract cancer	Baseline gut microbiota and metabolites predicted response and survival after PD-1/PD-L1 therapy.	*Alistipes*, *Bacilli*, *Lactobacillales*
Shaikh et al., 2024 [66]	Esophageal and gastroesophageal junction	Distinct baseline fecal microbiome and metabolite profiles were associated with pathological complete response following neoadjuvant ICIs plus chemoradiotherapy.	*Roseburia inulinivorans*, *Ruminococcus callidus*, *Fusicatenibacter saccharivorans*, *Bacteroides*.
Han et al., 2023 [67]	Gastric and gastroesophageal junction	Higher Lactobacillus abundance was associated with improved response to PD-1/PD-L1 immunotherapy.	*Lactobacillus, Ruminococcus*, *Eubacterium*, *Erysipelotrichaceae* (responders); *Streptococcus* spp. (non-responders).

**Table 2:** A summary of clinical studies evaluating the gut microbiome as a predictor of immunotherapy response in HCC.

Study	Population	Main Finding	Key Taxa	Association
Zheng 2019 [17]	China (*n* = 8)	Higher microbial diversity and early beta-diversity divergence associated with response	*Akkermansia muciniphila*, *Ruminococcaceae*	Yes
Lee 2022 [48]	Taiwan (*n* = 69)	No alpha-diversity difference but distinct beta-diversity separation	*Veillonella* *Lachnoclostridium* *Lactobacillales*	Yes
Zhu 2024 [69]	China (*n* = 80)	No alpha-diversity association; transient beta-diversity differences; distinct taxa identified between DCB and NDB	*Escherichia coli* *Phascolarctobacterium* *faecium* *Saccharibacteria*	Yes
Chung 2021 [72]	Korea (*n* = 8)	Higher alpha diversity and distinct beta clustering in responders; enrichment of SCFA-producing bacteria	SCFA-producing taxa (e.g., *Lachnospiraceae*, *Ruminococcaceae*, *Prevotella*, *Roseburia*) *Akkermansia*	Yes
Ponziani 2022 [68]	Italy (*n* = 11)	No diversity association with response; Akkermansia enriched in responders but no consistent non-responder taxa	*Akkermansia*	No
Shen 2021 [73]	Taiwan (*n* = 36)	No alpha or beta-diversity differences; disease control associated with specific beneficial taxa	*Bifidobacterium* *Coprococcus* *Acidaminococcus*	No
Inukai 2024 [71]	Japan (*n* = 37)	No diversity differences; distinct taxa profiles between responders and non-responders	*Tannerellaceae* *Parabacteroides* (incl. *Bacteroides stercoris*, *Parabacteroides merdae*)	No
Xin 2024 [70]	China (*n* = 45)	No alpha-diversity difference but significant beta-diversity separation; specific taxa associated with survival outcomes	*Collinsella* *Ruminococcus* spp.	Yes

**Table 3:** Current roles of biomarkers in HCC: strengths and limitations.

Biomarker Category	Specimen	Main Strengths	Main Limitations	Current Role in HCC
PD-L1 expression	Tumor tissue	Direct assessment of immune-related tumor phenotype; clinically established assays	Spatial heterogeneity, assay variability, limited predictive discrimination	Investigational; not sufficient for routine treatment selection
Tumor mutational burden	Tumor tissue or plasma	Genomic assessment of potential immunogenicity	Platform and threshold variability; limited HCC-specific validation	Investigational
Circulating biomarkers	Blood	Minimally invasive; suitable for longitudinal monitoring	Biological and assay heterogeneity; limited prospective validation	Investigational
Inflammatory indices	Routine blood tests	Inexpensive, accessible, reproducible	Non-specific; frequently prognostic rather than predictive	Prognostic enrichment and research use
Microbiome-derived signatures	Stool	Non-invasive; longitudinal; captures host–environment and immune–metabolic interactions	Major pre-analytical, analytical, geographic, dietary, medication-related, and cirrhosis-related variability	Exploratory; potential component of composite models

## Data Availability

No new data were created or analyzed in this study. Data sharing is not applicable to this article.

## Abbreviations

The following abbreviations are used in this manuscript:

CD	cluster of differentiation
cDC1	conventional type 1 dendritic cell
CTLA-4	cytotoxic T-lymphocyte-associated protein 4
CXCL16	C-X-C motif chemokine ligand 16
DESS	dimethyl sulfoxide + ethylenediaminetetraacetic acid + saturated NaCl
FMT	fecal microbiota transplantation
GPR43	G protein-coupled receptor 43
HCC	hepatocellular carcinoma
HMP	Human Microbiome Project
ICIs	immune checkpoint inhibitors
IDO	indoleamine 2,3-dioxygenase
IFN	interferon
IHMS	Human Microbiome Standards
IL	interleukin
ILC3s	group 3 innate lymphoid cells
isoDCA	iso-deoxycholic acid
JAK1	Janus kinase 1
MAMPs	microbial-associated molecular patterns
MAPK	mitogen-activated protein kinase
MyD88	myeloid differentiation primary response 88
NAFLD	non-alcoholic fatty liver disease
NF-κB	nuclear factor kappa-light-chain-enhancer of activated B cells
NK	natural killer
NLRP3	NOD-, LRR- and pyrin domain-containing protein 3)
PAMPSs	pathogen-associated molecular patterns
PD-1	programmed cell death protein 1
PD-L1	programmed death-ligand 1
PPI	proton pump inhibitors
PRRs	pattern recognition receptors
RORγt	retinoic acid receptor-related orphan receptor gamma t
RXR	farnesoid X receptor
SCFAs	short-chain fatty acids
STAT3	signal transducer and activator of transcription 3
TGF-β	transforming growth factor beta
TGR5	Takeda G protein-coupled receptor 5
Th1	T helper 1
TLRs	Toll-like receptors
TME	tumor microenvironment
TNF-α	tumor necrosis factor alpha
TRIF	Toll/interleukin-1-domain-containing adapter-inducing interferon-β
VSL#3	multi-strain probiotic formulation
